# Baicalein Induces Apoptosis of Rheumatoid Arthritis Synovial Fibroblasts through Inactivation of the PI3K/Akt/mTOR Pathway

**DOI:** 10.1155/2022/3643265

**Published:** 2022-09-07

**Authors:** Xue Zhang, Xia Guan, Yingshi Piao, Xiangguo Che, Mengge Si, Jingchun Jin

**Affiliations:** ^1^Department of Rheumatology, Affiliated Hospital of Yanbian University, Yanji 133002, Jilin Province, China; ^2^Department of Kidney Disease & Rheumatology, Tongliao City Hospital, Tongliao 028000, Inner Mongolia Autonomous Region, China; ^3^Laboratory of Cancer Research Center, Yanbian University Medical College, Yanji 133002, Jilin Province, China; ^4^Department of Biochemistry and Cell Biology, Cell and Matrix Research Institute, Korea Mouse Phenotyping Center, School of Medicine, Kyungpook National University, Daegu, Republic of Korea

## Abstract

**Purpose:**

Rheumatoid arthritis (RA) shows abnormal proliferation, apoptosis, and invasion in fibroblast-like synoviocytes (FLSs). Baicalein (BAI), extracted from *Scutellaria baicalensis*, is used as an anticancer drug through inducing cancer cells apoptosis. However, the mechanism of BAI in RA progression still remains unknown. Here, we demonstrated that BAI inhibited FLS proliferation and migration, whereas it enhanced apoptosis via the PI3K/Akt/mTOR pathway *in vitro*.

**Methods:**

Cell viability and colony formation were analyzed by MTT and plate colony formation assays in SW982 cells, respectively. Apoptosis was detected by flow cytometry and western blotting. Epithelial-mesenchymal transition (EMT), MMP family proteins (MMP2/9), and the PI3K/Akt/mTOR pathway were detected by western blot. Cell migration was detected by scratch healing assay under BAI treatment in SW982 cells.

**Results:**

BAI dose-dependently inhibited cell viability and colony forming in SW982 cells. BAI upregulated apoptotic proteins and downregulated EMT-related proteins, resulting in enhanced cell apoptosis and inhibited cell migration in SW982 cells. BAI also dose-dependently inhibited the phosphorylation of PI3K, Akt, and mTOR.

**Conclusions:**

These results indicated that BAI inhibited FLSs proliferation and EMT, whereas induced cell apoptosis through blocking the PI3K/Akt/mTOR pathway, supporting clinical application for RA progression.

## 1. Introduction

Rheumatoid arthritis (RA) is an autoimmune and systemic inflammatory disease that affects 0.5%–1% of the global population [1]. The basic pathological manifestations of rheumatoid arthritis are synovial inflammation, pannus formation, and progressive destruction of articular cartilage and bone, resulting in joint deformity and loss of function. RA shows abnormal proliferation, apoptosis and invasion in Fibroblast-like synoviocytes (FLSs). FSL activation induced chemokine secretion and immune cell recruitment to destroy bone and cartilage in the inflammatory environment of the synovium tissues [2–4]. To date, nonsteroidal anti-inflammatory drugs (NSAIDs), corticosteroids and disease-modifying antirheumatic drugs (DMARDs) are commonly used for RA treatment [5]. However, most of the drugs for RA patients' treatment have gastrointestinal side effect or are very difficult to treat for a long time because of their high cost. Therefore, development of a novel therapy for RA which has low cost, is effective, and has less side effects is very important [6]. New studies and reports have confirmed that extracts or active components of flavonoids, alkaloids, and triterpenoids of traditional Chinese herbal medicine have anti-RA effect [7].

Baicalein (BAI) (5,6,7-trihydroxyflavone) (Figure S1(a)) [8, 9], a traditional Chinese herbal medicine, extracted from *Scutellaria baicalensis,* is widely used as an anti-inflammatory or anticancer drug. *Scutellaria baicalensis* has been applied in the treatment of respiratory infection, diarrhea, jaundice, and hepatitis in traditional Chinese medicine [10]. In previous studies, we have demonstrated that BAI could inhibit gastric cancer cell proliferation and migration through FAK interaction via downregulation in AKT/mTOR signaling [11]. BAI also inhibited cell proliferation via blocking of the mTOR/p70S6K pathway and suppressed EMT pathway-associated migration through the TGF*β* pathway in cervical cancer HeLa cells [12]. According to network protein analysis of molecular docking system (Figure S1(b)), recent reports also found that BAI highly binding affinity to AKT1, MAPK1, MMP2, and MMP9 which is involved in cell proliferation apoptosis and migration [13].

Recently, studies have also shown that BAI can treat osteoarthritis (OA) by inhibiting chondrocyte apoptosis, reducing cartilage damage, and reducing synovitis, suggesting that BAI has the potential to slow the pathological progression of OA [14]. BAI significantly suppressed TNF-*α*, IL-6, and IL-1*β* mRNA expression in FLSs, demonstrating a potential therapeutic effect for RA through anti-inflammatory action [15]. Clinical investigation reveals a strong association RA progression with increased FLSs proliferation and migration, whereas inhibited FLSs apoptosis. However, the mechanism of BAI in RA progression still remains unknown. In this study, we found BAI inhibits FLS proliferation and EMT, whereas it induces cell apoptosis through blocking the PI3K/Akt/mTOR pathway.

## 2. Materials and Methods

### 2.1. Cells and Reagents

SW982 cell line was kindly supplied from the Cancer Research Center of Yanbian University. BAI was purchased from Cayman Chemical (Ann Arbor, Michigan, USA) and Annexinv-FITC/PI Apoptosis Detection Kit was purchased from BD Bioscience (Franklin Lakes, Jersey, USA).

### 2.2. Cell Proliferation Assay

Cell viability was determined by an MTT (3-(4,5-dimethylthiazol-2-yl)-2,5- diphenyltetrazolium bromide) assay. SW982 cells (5 × 10^4^ cells/ml) were cultured on 96-well plates with different concentrations of BAI (0 *μ*M, 15 *μ*M, 25 *μ*M, 50 *μ*M, 75 *μ*M, and 100 *μ*M) for 24, 48, 72, and 96 hours. After BAI treatment, 20 *μ*L of MTT solution (5 mg/mL) was added to each well and incubated for 4 hours at 37°C in a 5% CO_2_/95% air atmosphere. Precipitated formazan was dissolved in DMSO then detect absorbance at 490 nm by a spectrophotometric microplate reader (TECAN-infinite M200 pro, Mannersdorf, Switzerland).

### 2.3. Colony Formation Assay

Cells were plated at a density of 200 cells/well on 6 well plate with different concentrations of BAI for 14 days. Cells were fixed with anhydrous methanol for 30 minutes and stained with Giemsa Stain solution for 30 min at room temperature. The number of cell clones were counted under the microscope (>50 clones validated; Leica, Germany), and 3 times independent experiments were performed.

### 2.4. Cell Apoptosis Assay

The percentage of apoptotic cells were determined using FITC Annexin V Apoptosis Detection kit II (BD Bioscience, USA) and all the experiment steps were performed according to manufacturer's instructions. SW982 cells (1 × 10^6^ cells/well) were incubated with different concentrations of BAI for 48 hours and collected in the 15 ml conical tube and then washed twice with cold PBS. Cells were then resuspended in a 1X binding buffer (0.1 M HEPES/NaOH, pH7.4, 1.4 M NaCl, 25 mM CaCl_2_) and 1 × 10^5^ cells were transferred to 5 ml flow cytometry (FACS) tubes. FITC Annexin V (5 *μ*l) and propidium iodide (5 *μ*l) were added and the cells were incubated for 15 minutes at room temperature under a dark condition. The cells were analyzed by FACS advantage equipment (BD Bioscience, USA).

### 2.5. Wound Healing Assay

SW982 cells were plated at a density of 5 × 10^4^ cells/well on 24 well plate and were incubated for 24 hours upon reaching a confluent monolayer. Artificial wounds were formed by scratching with a 200 *μ*l pipette tip. The scratch-wounded cells were washed with 1 X PBS to remove cell fragments and were incubated in a fresh media with various concentrations of BAI. Three independent images of scratch-wounded region of interest (ROI) were captured by using a digital camera (Olympus, Japan) at 0, 24, 48 and 72 hours after BAI treatment.

### 2.6. Western Blotting

SW982 cells were cultured with different concentrations of BAI for 48 hours. Cell was lysed with RIPA buffer (50 mM Tris-HCl, pH7.4, 150 mM NaCl, 1 mM EDTA, 1% NP-40, 0.1% SDS, 0.5% sodium deoxycholate, 1 mM Na_3_VO_4_, and a protease inhibitor cocktail; Sigma-Aldrich). Cell lysates were measured Bradford protein assay kit (Bio-Rad) and were loaded on 8% or 10% polyacrylamide gels and electrophoresed in SDS-PAGE. Proteins were transferred to a PVDF membrane (Millipore, Eschborn, Germany) and then was blocked with 5% skim milk and immunoblotted with a primary antibody (Cell Signaling Technology, USA). Proteins were detected by enzyme-linked chemiluminescence (ECL) and quantified with a chemiluminescence and fluorescence imaging system (Bio-Rad, USA).

### 2.7. Statistical Analysis

All data analyses were performed using SPSS 26.0 software (SPSS, Chicago, IL) and GraphPad Prism 9.1.2 software (GraphPad Software, USA). The least significant difference (LSD) *t* test was used to compare two groups and one-way ANOVA was used for multiple comparisons. The value of *P* < 0.05 was considered statistically significant. Average values of at least 3 independent experiments were used for analysis.

## 3. Results

### 3.1. BAI Inhibited the Proliferation and Colony Formation of SW982 Cells

**Figure 1:**
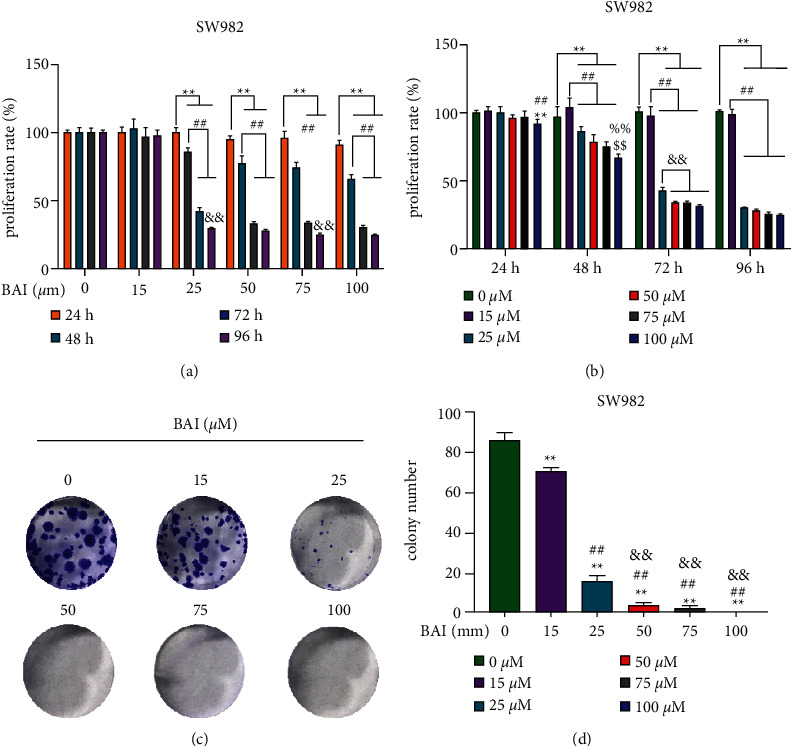
Negative regulation of BAI on the proliferation and colony formation in SW982 cells. (a), (b) Proliferation of human synovial sarcoma cell SW982 was determined by MTT assay, *n* = 6. (c) The colony-forming ability of human synovial sarcoma SW982 cells was detected by plate clonal formation assay, *n* = 3. (d) Quantitative data for (c). ^*∗∗*^*P* < 0.01, vs. 24 hours; ^##^*P* < 0.01, vs. 48 hours; ^&&^*P* < 0.01, vs. 72 hours; ^*∗∗*^*P* < 0.01, vs. 0 *μ*M (Control group); ^##^*P* < 0.01, vs. 15 *μ*M BAI group; ^&&^*P* < 0.01, vs. 25 *μ*M BAI group; ^%%^*P* < 0.01, vs. 50 *μ*M BAI group; ^$$^*P* < 0.01, vs. 75 *μ*M BAI group.

BAI inhibited the proliferation and growth of human osteosarcoma cells [16]; however, the effect on the proliferation of FSLs was not elucidated. MTT assay was used to detect the effects of BAI on SW982 cell viability and proliferation (Figures 1(a) and 1(b)). BAI dose-dependently and time-dependently inhibited SW982 cells proliferation at the concentration of 25 *μ*M, 50 *μ*M, 75 *μ*M, and 100 *μ*M. In addition, we also carried out plate cloning forming assay to detect effect of BAI on SW982 cells viability (Figures 1(c) and 1(d)). BAI significantly inhibited colony formation ability of SW982 cells at the concentration of 25 *μ*M, 50 *μ*M, 75 *μ*M, and 100 *μ*M. These results suggested that BAI could significantly inhibit cell proliferation and viability in human synovial sarcoma SW982 cells.

### 3.2. BAI Induced Apoptosis of SW982 Cells

**Figure 2:**
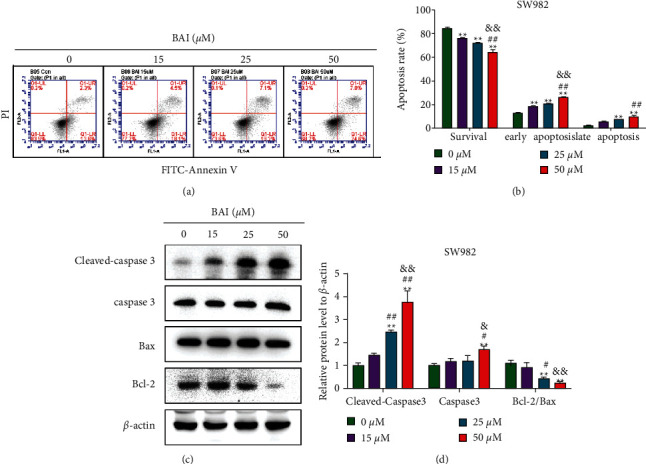
BAI promoted SW982 cell apoptosis. (a) Annexin V-FITC positive and PI-positive staining detected early apoptotic cells (lower right quadrant), Annexin V-FITC positive and PI-positive double staining detected late apoptotic cells (upper right quadrant), and Annexin V-FITC negative and PI-positive staining detected dead cells (upper left quadrant). (b) Quantitative data for (a). (c) Apoptosis-related proteins in SW982 cells were detected by western blotting. (d) Quantitative data for (c). ^*∗∗*^*P* < 0.01, vs. 0 *μ*M BAI group; ^#^*P* < 0.05 and ^##^*P* < 0.01, vs. 15 *μ*M BAI group; ^&^*P* < 0.05 and ^&&^*P* < 0.01, vs. 25 *μ*M BAI group. *n* = 3.

Enhanced proliferation and inhibited apoptosis in FLS are generally considered as a major pathological reason for RA development. However, rheumatoid arthritis FLSs (RA-FLSs) are characterized by apoptosis resistance, increased invasiveness, and the production of inflammatory mediators. BAI-treated groups at 15 *μ*M, 25 *μ*M, and 50 *μ*M promoted apoptosis of SW982 cells in a dose-dependent manner compared to the control group by FITC/PI double staining flow cytometry (Figures 2(a) and 2(b)). Expression of cleaved-caspase3, caspase3, and Bax expression were dose-dependently increased while the Bcl-2 expression was dose-dependently decreased at BAI treatment in the SW982 cells (Figures 2(c) and 2(d)). These results indicated BAI could induce SW982 cells apoptosis.

### 3.3. BAI Inhibited the PI3K/Akt/mTOR Signaling Pathway

**Figure 3:**
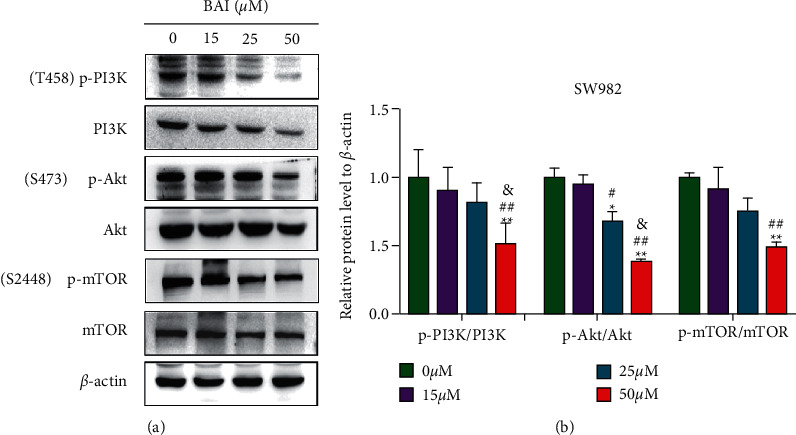
Regulation of BAI on the PI3K/Akt/mTOR signaling pathway in SW982 cells. (a) PI3K/Akt/mTOR signaling pathway-associated proteins were detected by western blotting in BAI-treated SW982 cells. (b) Quantitative data for (a). ^*∗*^*P* < 0.05 and ^*∗∗*^*P* < 0.01, vs. 0 *μ*M BAI group; ^#^*P* < 0.05 and ^##^*P* < 0.01, vs. 15 *μ*M BAI group; ^&^*P* < 0.05 and ^&&^*P* < 0.01, vs. 25 *μ*M BAI group. *n* = 3.

The PI3K/Akt/mTOR signaling pathway is an important signaling pathway in the cell proliferation and apoptosis. To elucidate the molecular mechanism of BAI-induced apoptosis, we confirmed the PI3K/Akt/mTOR signaling pathway. Interestingly, BAI inhibited the expression of p-PI3K, p-Akt, and p-mTOR, while the expression of PI3K, Akt and mTOR did not change significantly (Figures 3(a) and 3(b)). These results indicated BAI could dose-dependently inactivate of PI3K/Akt/mTOR signaling pathway to regulate SW982 cells apoptosis.

### 3.4. BAI Inhibited Migration of SW982 Cells

**Figure 4:**
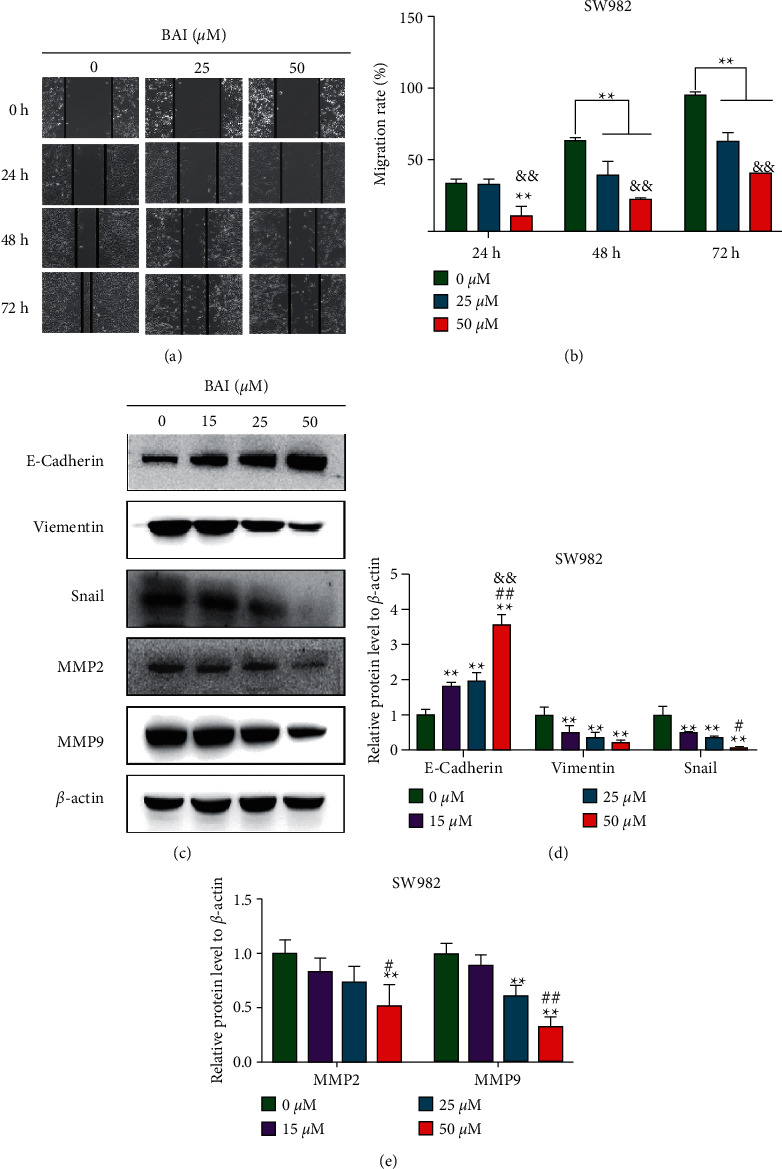
BAI inhibited the SW982 migration. (a) Cell migration ability was performed by cell scratch assay. (b) Quantitative data for (a). (c) SW982 cells were treated with BAI at 0 *μ*M, 15 *μ*M, 25 *μ*M, and 50 *μ*M for 48 h, and the protein expression was detected by western blotting. (d, e) Quantitative data for (c). ^*∗∗*^*P* < 0.01, vs. 0 *μ*M BAI group; ^#^*P* < 0.05 and ^##^*P* < 0.01, vs. 15 *μ*M; ^&&^*P* < 0.01, vs. 25 *μ*M. *n* = 3.

RA-FLSs secrete inflammatory cytokines, chemokines, and MMPs and induce cell migration and invasion to destroy joints. Therefore, the regulation of RA-FLS migration may be a new therapeutic strategy for the pathologic progression of RA [17]. Due to migration of FLSs involved in the RA development and progression, we further examined the effect of BAI on SW982 cell migration through a scratch healing experiment. Lateral migration of SW982 cells was significantly reduced in the BAI-treated group (25 *μ*M and 50 *μ*M) at 24, 48, 72, and 96 hours (Figures 4(a)–4(b)). BAI treatment significantly upregulated epithelial markers of E-cadherin in a dose-dependent manner compared with controls, whereas it dose-dependently downregulated epithelial markers of vimentin, snail, MMP2, and MMP9 (Figures 4(c)–4(e)). These results suggested that BAI significantly inhibited the lateral and vertical migration of SW982 cells via regulation of epithelial-mesenchymal transformation (EMT)-related proteins and MMP family proteins expression.

## 4. Discussion

RA is a chronic inflammatory disease characterized by autoimmunity, infiltration of activated inflammatory cells into the synovial membrane of the joint, synovial proliferation, new angiogenesis, and progressive destruction of cartilage and bone; the main pathological change is chronic inflammation of synovial tissue [18, 19]. The synovial environment of RA is conducive to the survival of FLSs and inhibits its apoptosis, thereby participating in preventing its elimination. The imbalance of FLS proliferation and apoptosis has been considered as an initiation factor in RA development [20, 21]. Therefore, the development of a novel therapy for RA which inhibits antiproliferation and induces apoptosis is a very important approach. BAI inhibits cell proliferation via blocking of the mTOR/p70S6K pathway and suppresses EMT pathway-associated migration through the TGF*β* pathway in cervical cancer HeLa cells [12]; however, the effect on the mechanism of BAI on RA development and progression still remained unknown. The human synovial sarcoma cell line SW982 was one of the well-known models for synovitis in RA [22]; therefore, we used human synovial sarcoma cell line SW982 to determine mechanism of BAI in RA development and progression.

Chinese herbal medicine is considered as a valuable resource for novel drug candidates with low toxicity. A large number of studies have confirmed that Chinese herbs and their components have proapoptotic activities on FLSs. Zhang et al. reviewed these Chinese herbs and pointed out that inducing apoptosis of FLSs is an important molecular mechanism of the active components of Chinese herbs in the treatment of rheumatoid arthritis [6].

BAI is a traditional Chinese herbal medicine with anti-inflammatory and antioxidant activities [23, 24]. However, the therapeutic effect of BAI on RA is not fully understood [25]. Tang et al. found that BAI may be a candidate drug for treatment of RA and the biological function of the grouping network related to the pathogenesis of RA is clarified. Including some viral infections and cancers, involving related signaling pathways such as the PI3K-Akt signaling pathway [26], Hu et al. found that BAI inhibited cervical cancer cell growth and cell cycle whereas enhanced cell apoptosis [27]. Recent reports demonstrated that BAI regulated Bax, Bcl-2, and caspase3 apoptosis signaling pathways to protect OA development [14].

Synovial hyperplasia and infiltration of immune cells lead to excessive expansion and destruction of articular cartilage in RA [28]. Activated FLSs are resistant to cell apoptosis. Indeed, activated FLSs induce chemokine secretion to promote inflammation, and neovascularization ultimately accelerate cartilage degradation [29, 30]. Small molecule inflammatory mediators and proteolytic enzymes produced by FLSs can degrade the extracellular matrix in RA [31]. However, simple TNF-*α* blockade could not directly kill inflammatory FLSs under the microenvironment of RA patients. Direct targeting of inflammatory FLSs to enhance apoptosis could be an effective therapeutic strategy for RA treatment [32]. In this study, we found that BAI enhanced the apoptosis of SW982 cells while inhibited the proliferation of SW982 cells, which might provide a new basis for the treatment of RA.

**Figure 5:**
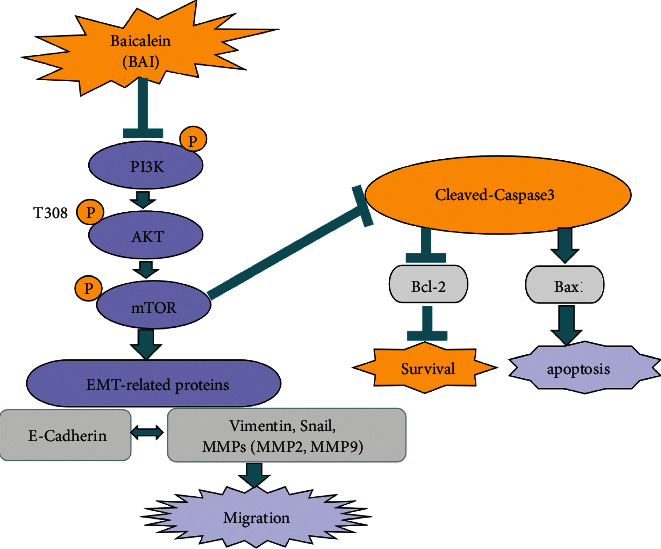
Schematic diagram of BAI-induced apoptosis and inhibited cell proliferation and migration. BAI inhibited cell proliferation by regulating PI3K/Akt/mTOR and activated cleaved caspase 3 to induce apoptosis and also inhibited cell migration.

The PI3K/Akt/mTOR pathway regulates cell proliferation and migration; therefore, its role in RA synovial hypertrophy and immune cell infiltration has been extensively explored. The PI3K/Akt/mTOR signaling pathway is activated in a variety of RA cell lines [33–35]. In synovial cells of RA patients, the signaling pathway is continuously abnormally activated, resulting in a high expression of antiapoptotic genes [36]. The PI3K/Akt/mTOR signaling pathway was significantly activated in RA synovial cells and attenuated RA development by modulating the PI3K/Akt/mTOR signaling pathway [37]. Thus, inhibition of the PI3K/Akt signaling pathway mediated an antiapoptotic action, which is the main therapeutic target for RA treatment [38]. Recent relevant studies reported that inactivation of the PI3K/Akt/mTOR signaling pathway enhanced autophagy, inhibited the proliferation and inflammatory response of synovial cells, and thus alleviated local symptoms of RA [39]. In addition, studies have also shown that galangin inhibited the development of RA through the PI3K/AKT signaling pathway, which also revealed a new mechanism which might be a feasible method for the treatment of RA [40]. Recent studies showed that BAI not only inhibited nasopharyngeal carcinoma (NPC) cells proliferation and induced NPC cells apoptosis but also blocked the PI3K/AKT and p53 signaling pathways in CNE2 cells [41]. Our studies want to clarify the molecular mechanism of BAI-induced apoptosis, and the relation with the PI3K/Akt/mTOR signaling pathway in RA-FLSs (Figure 5). We confirmed BAI inhibited cell proliferation and reduced p-PI3K, p-Akt, and p-mTOR expression compared to the control group in SW982 cells.

In addition, EMT is a key process in tumor metastasis and is characterized by decreased expression of intercellular adhesion molecules such as E-cadherin and increased expression of mesenchymal proteins such as vimentin. During EMT, epithelial cells adopt a mesenchymal phenotype and show increased migration potential, thus increasing invasiveness and resistance to apoptosis [42, 43]. Snail induces EMT through the suppression of many epithelial markers, including E-cadherin and claudins [44, 45]. E-cadherin is one of the molecular markers of the epithelial phenotype, downregulation or loss of E-cadherin leads to the breakdown of intercellular connections and promotes cell metastasis, which is the most significant event during EMT [46]. Ma et al. reported BAI significantly downregulated snail and vimentin protein, upregulated E-cadherin protein in a dose- and time-dependent manner on breast cancer [47]. Similarly, in our study, BAI was also proved to inhibit the occurrence of EMT by downregulated snail and vimentin proteins, upregulated the E-cadherin protein thus inhibiting the migration of SW982 cells.

The migration and invasion of RA-FLSs play an important role in synovitis and bone destruction [48]. Activated RA-FLSs not only migrate and invade adjacent unaffected joints but also migrate to the sublayer of synovial lining, promoting angiogenesis, and exacerbating synovial hyperplasia and bone destruction [49]. Recently, some scholars confirmed that miR-653-5p inhibited the cell migration and invasion in RA-FLS cells and revealed that regulating the migration and invasion of RA-FLSs might be a new strategy for RA treatment [50]. It is well known that MMPs, mainly produced by FLSs in RA, are proteases involved in extracellular matrix remodeling and play an important role in FLSs migration and progressive joint destruction in RA. Rui et al. found that BAI significantly inhibited the invasive and metastatic ability of colorectal cancer cells by regulating the expression levels of MMP2/9 via inhibition of the AKT signaling pathway [51]. In this paper, we further confirmed that BAI could inhibit the migration of SW982 cells by a cell scratch healing experiment, and its migration mechanism might be related to the downregulation of the MMP2/9 expression, EMT-related proteins, and PI3K/Akt/mTOR signaling pathway.

## 5. Conclusion

In summary, BAI inhibited FLS proliferation and EMT, whereas induced cell apoptosis through blocking the PI3K/Akt/mTOR pathway, supporting clinical application for RA progression. The abovementioned studies indicated the possibility of BAI as a candidate drug for the treatment of RA in the future and provided a solid data and theoretical basis for its application in clinical treatment. Of course, more research could be needed to confirm this, and this will be the focus of our future research.

## Data Availability

The data used to support the findings of this study are available from the corresponding author upon request.

## Supplementary Materials

Supplementary Materials Supplementary Figure S1: (a) Structures of baicalein (BAI). (b) Diagram of BAI/AKT1 protein STRING interaction network. The interaction between BAI and related proteins was detected by online network protein analysis of string database, and BAI may be related to MMP2 and MMP9 proteins, as well as AKT1 proteins.
