# Supplementary material for: Baicalein Induces Apoptosis of Rheumatoid Arthritis Synovial Fibroblasts through Inactivation of the PI3K/Akt/mTOR Pathway
Source: Evid Based Complement Alternat Med. 2022 Sep 7;2022:3643265. doi: 10.1155/2022/3643265 (PMC9473868; doi:10.1155/2022/3643265)
Supplement: Supplementary Materials — Supplementary Figure S1: (a) Structures of baicalein (BAI). (b) Diagram of BAI/AKT1 protein STRING interaction network. The interaction between BAI and related proteins was detected by online network protein analysis of string database, and BAI may be related to MMP2 and MMP9 proteins, as well as AKT1 proteins. [file 3643265.f1.doc]

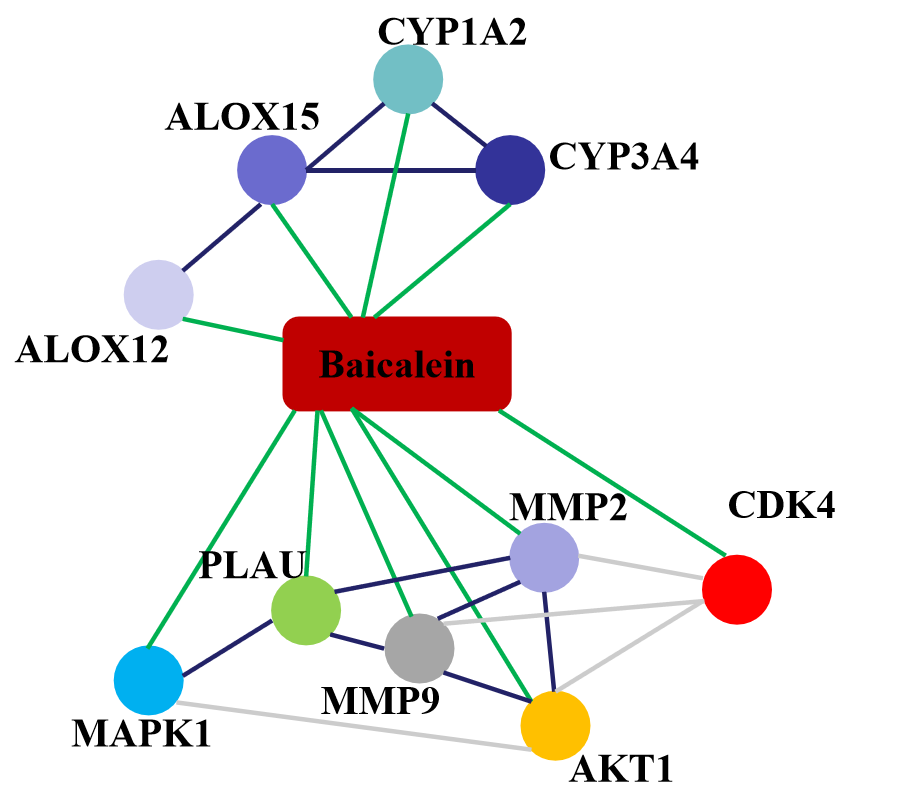


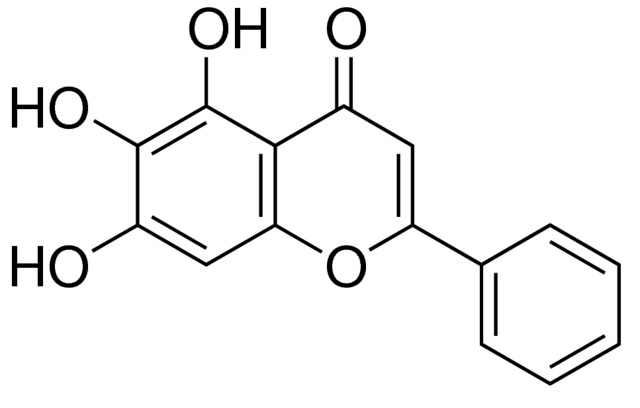


**Baicalein**

1. (a)
2. (b)

Figure S1: (a)Structures of baicalein (BAI). (b) Diagram of BAI/AKT1 protein STRING interaction network.
